# The Replicative DnaE Polymerase of *Bacillus subtilis* Recruits the Glycolytic Pyruvate Kinase (PykA) When Bound to Primed DNA Templates

**DOI:** 10.3390/life13040965

**Published:** 2023-04-07

**Authors:** Alexandria Holland, Matthaios Pitoulias, Panos Soultanas, Laurent Janniere

**Affiliations:** 1 Biodiscovery Institute, School of Chemistry, University of Nottingham, Nottingham NG7 2RD, UK; 2 Génomique Métabolique, Genoscope, Institut François Jacob, CEA, CNRS, Université Evry, Université Paris-Saclay, 91057 Evry, CEDEX, France

**Keywords:** microbiology, metabolism, replication, PykA, DnaE, moonlighting activity

## Abstract

The glycolytic enzyme PykA has been reported to drive the metabolic control of replication through a mechanism involving PykA moonlighting functions on the essential DnaE polymerase, the DnaC helicase and regulatory determinants of PykA catalytic activity in *Bacillus subtilis*. The mutants of this control suffer from critical replication and cell cycle defects, showing that the metabolic control of replication plays important functions in the overall rate of replication. Using biochemical approaches, we demonstrate here that PykA interacts with DnaE for modulating its activity when the replication enzyme is bound to a primed DNA template. This interaction is mediated by the CAT domain of PykA and possibly allosterically regulated by its PEPut domain, which also operates as a potent regulator of PykA catalytic activity. Furthermore, using fluorescence microscopy we show that the CAT and PEPut domains are important for the spatial localization of origins and replication forks, independently of their function in PykA catalytic activity. Collectively, our data suggest that the metabolic control of replication depends on the recruitment of PykA by DnaE at sites of DNA synthesis. This recruitment is likely highly dynamic, as DnaE is frequently recruited to and released from replication machineries to extend the several thousand RNA primers generated from replication initiation to termination. This implies that PykA and DnaE continuously associate and dissociate at replication machineries for ensuring a highly dynamic coordination of the replication rate with metabolism.

## 1. Introduction

Pyruvate kinase is the enzyme responsible for the final stage of glycolysis, catalyzing the formation of pyruvate and ATP from phosphoenolpyruvate (PEP) and ADP. Mammals contain four isoforms of pyruvate kinase, whereas most bacteria contain a single isoform. The mammalian isoform PKM2 and some bacterial isoform PykA exhibit non-metabolic roles in addition to their metabolic functions. Mammalian pyruvate kinase has been shown to regulate transcription by phosphorylating transcription factors or binding to transcription factors to enhance gene expression levels [1,2]. Jannière et al. first observed in *Bacillus subtilis* the suppression of temperature-sensitive mutations in genes coding for some replication proteins by the dysfunction of certain genes coding for glycolytic enzymes [3]. Particularly, mutants in *pgm, pgk, eno*, and *pykA* genes reversed the phenotypes of mutations in genes coding for DnaE (the lagging strand DNA polymerase), DnaC (the replicative helicase—the homologue of *Escherichia coli* DnaB), and DnaG (the primase), strongly suggesting the existence of a genetic connection between glycolysis and DNA replication. Subsequently, PykA was shown to exhibit an important regulatory role linking metabolism to replication [4], and recently, PykA has been shown to typify a novel family of cross-species replication regulators that drive the metabolic control of replication through a mechanism involving regulatory determinants of PykA catalytic activity [5]. Surprisingly, the disruption of this regulatory control causes dramatic replication and cell cycle defects, showing that the metabolic control of replication is important for the overall rate of DNA synthesis [5]. As failures in replication control increase the risk of replication errors and double-stranded DNA breaks, dysregulation of the metabolic control of replication may pave the way for genetic instability and carcinogenesis [6,7,8,9].

**Figure 1:**
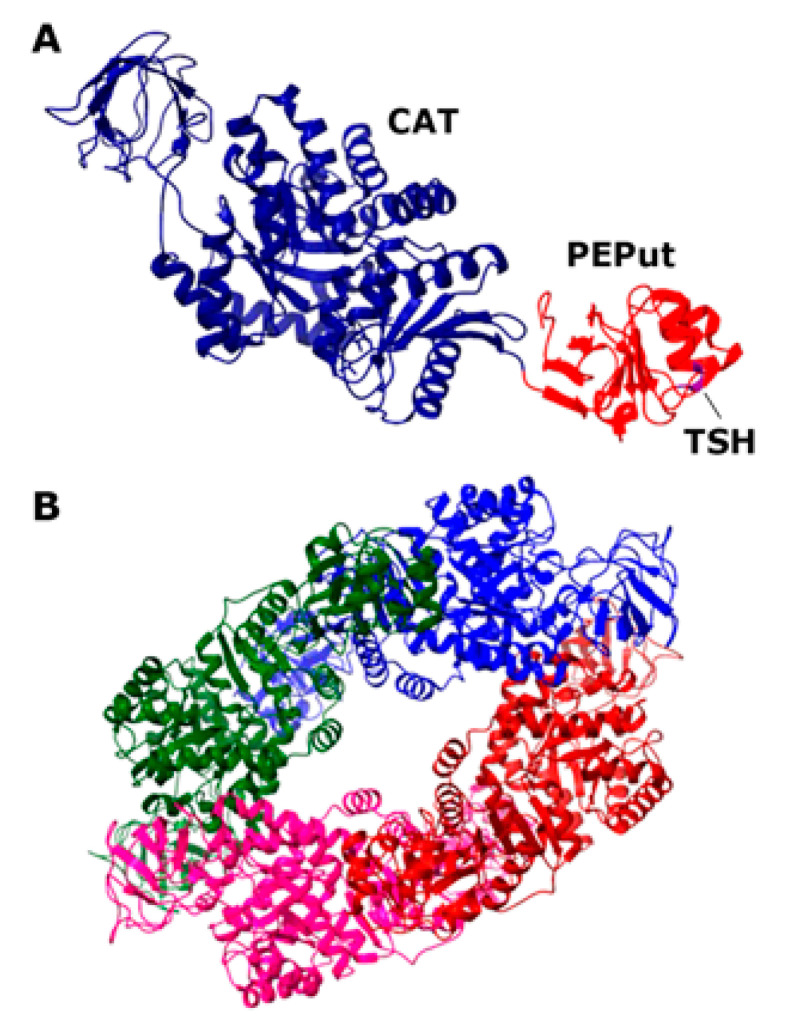
Structure of the *Geobacillus stearothermophilus* PykA protein. (**A**) Structure of the monomer (PDB2E28; 72.48% sequence identity to the *B. subtilis* PykA). The CAT and PEPut domains are colored blue and red, respectively, and the location of the TSH motif is shown. (**B**) Structure of the functional tetramer (PDB2E28). The four monomers are colored differently.

The mammalian PykA isoform PKM2 is a homotetramer that is affected by allosteric regulators, such as fructose 1,6-biphosphate (FBP) and serine, which promote tetramer assembly, while cysteine promotes tetramer dissociation [10,11,12]. The structure of the PykA tetramer from many organisms has been solved, highlighting the conservation of their global architecture and active site [13]. Almost all pyruvate kinases are homotropically activated by the substrate PEP and regulated by allosteric heterotropic effectors [13]. The allosteric regulation mechanism involves conformational changes between neighboring subunits around the tetramer, resulting in different structural states. In *Bacillus subtilis*, the heterotrophic effectors of PykA are AMP and ribose 5-phosphate [14], which appear to stabilize the active conformer [15]. Each monomeric subunit of the *Geobacillus stearothermophilus* PykA protein, which is 72.4% identical to *B. subtilis* PykA, comprises a catalytic domain (CAT, residues 1–473), containing the substrate and effector binding sites and an additional C-terminal domain with a structure resembling the PEP utilizer (PEPut, residues 474–586) domain of pyruvate phosphate dikinase and other metabolic enzymes (Figure 1) [16,17]. In these enzymes, PEPut is phosphorylated at a conserved TSH motif (residues 536–539) at the expense of PEP and ATP to drive sugar imports and catalytic or regulatory activities [18,19,20,21,22,23,24,25,26,27,28]. The PEPut domain is not required for the catalytic activity of PykA [16]. Instead, it has a regulatory role on the enzyme activity via its interaction with CAT and the phosphorylation status of its TSH motif [5]. PEPut interacts with CAT via a hydrogen bond between E209 and L536, assisting the heterotrophic effectors in stabilizing the active R-state conformation [15,29]. The CAT and PEPut domains were shown to exert different effects on DNA replication. CAT is a stimulator of elongation and PEPut is a nutrient-dependent inhibitor of initiation [5]. These activities depend on substrates binding to CAT, the CAT-PEPut interaction, and the phosphorylation status of the TSH motif. The direct functional effects of PykA on the activities of the DnaE and DnaC replication proteins were demonstrated with in vitro primer extension assays and helicase assays, respectively [5]. Despite the functional evidence of an interaction between PykA and the replication proteins DnaE and DnaC, there is no direct physical evidence of a protein–protein interaction. Here, we demonstrate a direct protein–protein interaction between PykA and DnaE, and further show that this interaction is mediated by CAT, depends on the binding of DnaE to primed DNA templates, and that PEPut somehow regulates the moonlighting function of PykA on DnaE activity. Overall, this study suggests that the metabolic control of replication depends on a dynamic recruitment of PykA at sites of DNA synthesis by DnaE and that the impact of this recruitment on DNA synthesis may be allosterically regulated by PEPut.

## 2. Materials and Methods

### 2.1. Cloning of the pykA Gene and Production and Purification of the Protein

#### 2.1.1. Cloning of *pykA*

A DNA fragment of 1755 bp carrying the *B. subtilis pykA* gene was amplified from genomic DNA using the PyKAF (5′-TACTTCCAATCCAATGCAAGAAAAACTAAAATTGTTTGTACCATCG-3′) and PykAR (5′-TTATCCACTTCCAATGTTATTAAAGAACGCTCGCACG-3′) forward and reverse primers, respectively, in colony PCR reactions using Q5 high-fidelity DNA polymerase according to supplier instructions. Typically, a *B. subtilis* single colony was suspended in 20 mL LB and grown at 37 °C until the optical density reached 0.4–0.8 at 600 nm. Thereafter, colony PCR reactions were carried out with genomic DNA (10 µL) acting as the template at a 10-fold dilution. The PCR reactions were carried out in a volume of 50 µL with one unit of Q5 high-fidelity DNA polymerase, 0.5 µM PykAF and PykAR, and 0.25 mM dNTPs in 1XQ5 polymerase buffer. PCR products were cleaned up with the Clean-up kit (Thermo Scientific, Waltham, MA, USA), resolved by agarose electrophoresis, and gel extracted using the GeneJET Gel Extraction kit (Thermo Scientific) and cloned into the p2CT plasmid (gift by James Berger) (Supplementary Figure S1) using ligation-independent cloning to construct the p2CT-*pykA* production vector which produces a terminally His-MBP-tagged PykA protein.

#### 2.1.2. Expression of *pykA*

For heterologous expression of the *B. subtilis pykA*, the p2CT-BsuPykA production vector was introduced into competent Rosetta (DE3) *E. coli* cells. Single colonies were used to inoculate two 600 mL 2xYT cultures, supplemented with 60 µL carbenicillin (50 mg/mL) in 2 L conical flasks. The flasks were incubated at 37 °C, with shaking (180 rpm) until the optical density reached 0.6–0.8 at 600 nm. Expression of *pykA* was induced by the addition of 0.5 mM IPTG and after further 3 h of growth, the cells were harvested by centrifugation at 3000× *g* for 15 min. Cells were suspended in 30 mL of buffer A (500 mM NaCl, 50 mM Tris-HCl pH 7.5, and 20 mM imidazole), supplemented with 1 mM PMSF and 50 µL protease inhibitor cocktail (Fischer, Waltham, MA, USA), and sonicated using a SANYO Soniprep 150 set at 15 amplitude microns 4 times for 1 min with 1 min intervals on ice in between. Then, benzonase (20 µL) was added to the cell lysate, which was further clarified at 40,000× *g* for 40 min. The clarified soluble crude extract was isolated and filtered through a 0.22 µm filter.

#### 2.1.3. Purification of PykA

The PykA protein was purified from the filtered crude extract using a combination of IMAC (Immobilized Metal Affinity Chromatography) and gel filtration. First, the filtered crude extract was loaded onto a 5 mL HisTrap HP column (GE Healthcare, Chicago, IL, USA) equilibrated in buffer A. The column was washed thoroughly with buffer A and the PykA protein was eluted using gradient elution with buffer B (500 mM NaCl, 50 mM Tris-HCl pH 7.5, and 1 M imidazole). The eluted PykA protein was collected and quantified spectrophotometrically (extinction coefficient 76,780 M^−1^ cm^−1^). TEV protease was added at 1:20 (TEV protease:PykA) molar ratio while dialyzing the protein solution overnight in dialysis buffer (500 mM NaCl and 50 mM Tris-HCl pH 7.5) at 4 °C in order to remove the His-MBP tag. The untagged PykA protein was then loaded back onto a 5 mL HisTrap HP column equilibrated in buffer A and the flow-through containing the untagged PykA was collected. Finally, the PykA protein solution was concentrated to 5–7 mL using a vivaspin 10 kDa cut-off filter. EDTA was added to 1 mM and the PykA preparation was then loaded onto a HiLoad 26/60 Superdex 200 Prep Grade gel filtration column (GE Healthcare) equilibrated in buffer C (500 mM NaCl, 50 mM Tris -HCl pH 7.5, and 1 mM EDTA). Fractions containing the PykA protein were pooled, and the purified PykA protein was quantified spectrophotometrically (extinction coefficient 8940 M^−1^ cm^−1^), aliquoted, and stored in −80 °C.

#### 2.1.4. PykA Activity Assay

The activity of purified PykA was assayed coupling the PykA catalyzed reaction (conversion of phosphoenolpyruvate to pyruvate) to the conversion of pyruvate into lactate catalyzed by LDH (Lactate Dehydrogenase) in the presence of NADH at 25 °C. The oxidation of NADH to NAD, which is directly proportional to the activity of PykA, was followed spectrophotometrically at 340 nm. The LDH-catalyzed reaction was first optimized to ensure that it did not become a limiting factor when measuring the activity of PykA. Then, PykA catalyzed reactions were carried out in 96-well plates using the reaction master mix 10 mM Tri-HCl pH 7.5, 10 mM MgCl_2_, 50 mM KCl, 0.5 mM NADH, 2 mM ADP, 9.375 × 10^−4^ mg/mL LDH, and 5.7 µg/mL PykA or CAT, at 25 °C. Using the GraphPad Prism 9 software, data were fit to an allosteric sigmoidal curve with reaction rates on the *Y*-axis and PEP concentration on the *X*-axis.

### 2.2. Cloning of a DNA Fragment Coding for the PEPut Domain of PykA and Production and Purification of the Peptide

#### 2.2.1. Fragment Cloning

The DNA fragment coding for the PEPut domain, with the preceding 10 amino acids, was isolated by PCR using genomic DNA and the pepF (5′-TACTTCCAATCCAATGCAGCACAAAATGCAAAAGAAGCT-3′) and pepR (5′-TTATCCACTTCCAATGTTATTAAAGAACGCTCGCACG-3′) primers. Typically, a *B. subtilis* single colony was suspended in 20 mL of LB and grown at 37 °C until the optical density reached 0.4–0.8 at 600 nm. Thereafter, PCR reactions were carried out with 10 μL of undiluted, 10× and 100× diluted samples in 1XQ5 polymerase buffer, 0.25 mM dNTPs, 0.5 μM primers (pepF and pepR), and Q5 polymerase (1 unit) in a total volume of 50 μL, using the following program: 1Xcycle 98 °C 5 min, 30Xcycles 98 °C 1 min, Ta (optimal annealing temperature) 30 s, 72 °C 30 s/kb, and 1Xcycle 72 °C 5 min. The resulting PEPut DNA fragment was cloned into the p2CT plasmid using ligation-independent cloning to produce the p2CT-PEPut production vector, in a process similar to that described above for the *pykA* (see also Supplementary Figure S1). The resulting p2CT-PEPut vector produced an N-terminally His-MBP-tagged PEPut protein. The His-MBP tag was removable by proteolysis using the TEV protease.

#### 2.2.2. Production and Purification of PEPut

Production and purification of the PEPut were carried out as described for the full-length PykA protein but the last gel filtration step during purification was omitted. Quantification of the final untagged PEPut (MW 9582.8 Da) was carried out spectrophotometrically using the extinction coefficients 69,330 M^−1^ cm^−1^ (for the His-MBP tagged PEPut) and 1490 M^−1^ cm^−1^ (for the untagged PEPut after TEV protease treatment).

### 2.3. Cloning of DNA Fragments Coding for CAT or Mutated PykA, Expression, and Purification of the Proteins

Template DNA of p2CT-BsuPykA plasmid containing the gene for wild-type PykA was used to generate the *pykA* mutants using site directed mutagenesis (NEB Q5 site directed mutagenesis kit) and the CAT construct. The primers listed in Table 1 were purchased from Sigma. Gene overexpression and purification of all mutated proteins and the CAT domain were carried out as described for the wild-type PykA protein.

### 2.4. Production and Purification of the B. subtilis DnaE Polymerase

The *B. subtilis dnaE* gene was cloned and the encoded protein was produced and purified, as described previously [30,31].

### 2.5. Polymerase Assays

Time-course polymerase assays were carried out by monitoring the DnaE primer extension activity using a 5′-end fluorescently labeled (Cy5; Sigma) 15 mer synthetic oligonucleotide (5′-AAGGGGGTGTGTGTG-3′) annealed onto a 110 mer oligonucleotide (5′-CACACACACACACACACACACACACACACACACACACACACACACACACACACACACCCCCTTTAAAAAAAAAAAAAAAAGCCAAAAGCAGTGCCAAGCTTGCATGCC-3′ in 50 mM Tris-HCL pH7.5, 50 mM NaCl, 10 mM MgCl_2_, 1 mM DTT, 1 mM dNTPs, and DnaE (4 nM) in the presence or absence of PykA, CAT, and PEPut (the concentrations of these proteins are specified in the relevant figure legends). The DNA products of the reactions were resolved with denaturing urea PAGE (15% gels, prepared and run in 1 XTBE). Visualization and image capturing were carried out using Licor Odyssey Fc Imaging System.

### 2.6. Surface Plasmon Resonance (SPR)

Initial SPR experiments were carried out in the absence of DNA, with only purified DnaE and PykA proteins. SPR experiments were carried out with a four-channel sensor C1 chip and a Biacore T200. The details of DnaE immobilization on the sensor chip surface, using EDC/NHS {1-ethyl-3(-dimethylaminopropyl)carboiimide hydrochloride/*N*-hydroxysuccinimide} chemistry, were as described elsewhere [26]. Subsequent experiments with DNA were carried out using a primed probe. Probes were constructed by annealing 30 mer (5′-AGGGGGTGTGTGTGTGTGTGTGTGTGTGTG-3′) to the 5′-end biotinylated 110 mer (5′-CACACACACACACACACACACACACACACACACACACACACACACACACACACACACACCCCCTTTAAAAAAAAAAAAAAAAGCCAAAAGCAGTGCCAAGCTTGCATGCC-3′). The annealing reaction comprised of the biotinylated 110 mer oligonucleotide and the 30 mer oligonucleotide template in slight molar excess (1.2:1), incubated at 95 °C for 2 min, and subsequently allowed to slowly cool to room temperature. Experiments were carried out at the Research Complex at Harwell in Oxford using a Biacore T200 at 200 μL/min with running buffer (25 mM NaCl, 25 mM MgCl_2_, 150 mM Tris-HCl pH 7.5, and 1 mM DTT) at 25 °C. 5′ biotinylated DNA probe was immobilized to a streptavidin-coated chip surface (Biacore SA chip) to yield an increase of approximately 100 response units (RUs) per flow cell. When required to remove proteins that were bound to immobilized DNA, flow cells were regenerated by washing with 0.5% *w*/*v* SDS.

### 2.7. B. subtilis Strains and Growth Conditions

*B. subtilis* strains are listed in Supplementary Table S1. They were constructed by transforming competent cells with genomic DNA. The genotypes of constructed strains were checked by phenotypic analyses, endonuclease restriction, PCR analysis, and/or Sanger sequencing (Eurofins Genomics, Germany). Routinely, *B. subtilis* cells were grown at 37 °C in LB plus malate (0.4% *w*/*v*) with or without antibiotics at the following concentrations: spectinomycin (Sp, 60 µg/mL); tetracycline (Tet, 7.5 µg/mL). Microscopy studies were carried out with cells grown at 37 °C in the MC medium [5].

### 2.8. Microscopy

Cells were first grown overnight at 30 °C in MC supplemented with antibiotics. Upon saturation, cultures were diluted 1000-fold in the same medium without antibiotics and growth at 37 °C was carefully monitored using spectrophotometry. At OD_600nm_ = 0.15, 1.5 mL of cells were centrifuged at room temperature (11,000 rpm for 1 min), resuspended in 10 µL MC medium, and immediately mounted onto a glass slide covered with a 1.0% *w*/*v* agarose pad in MC. A 0.17 mm glass coverslip was then placed on top of the agarose pad. Microscopy was carried out using an epifluorescence microscope (Zeiss, Axio Observer.Z1) with a 100× magnification oil-immersion objective (Plan-APOCHROMAT Ph3) and a CMOS camera (Orca Flash 4.0 LT Hamamatsu). Digital images were acquired and analyzed using the Zen 2.6 (blue edition) software.

## 3. Results

### 3.1. The CAT Domain Alone Elicits a Functional Effect on the DnaE Polymerase Activity and PEPut Modulates This Effect and PykA Catalytic Activity

**Figure 2:**
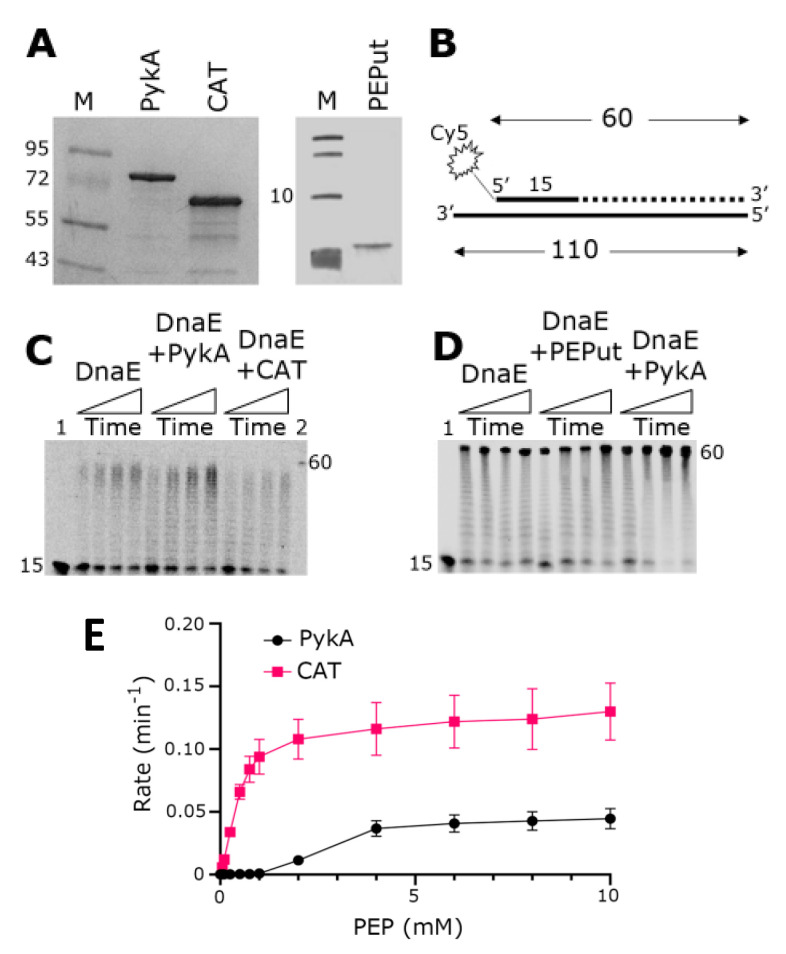
Replication and metabolic activities of purified PykA, CAT, and PEPut proteins. (**A**) SDS-PAGE gels showing purified PykA, CAT, and PEPut. Molecular weight markers in lanes M are shown in kDa units. (**B**) The radioactively labeled synthetic oligonucleotide probe used in primer extension assays is shown schematically. The 5′-end fluorescently labeled 15 mer oligonucleotide is annealed onto a 110 mer oligonucleotide. DnaE extends the 15 mer oligonucleotide to form a 60 mer product, as shown schematically by the dotted line. (**C**) Primer extension assays showing the biochemical effects of PykA and CAT on the DnaE polymerase activity. Time-course reactions (30, 60, 90, and 120 s, represented schematically from left to right by the rectangular triangles) were carried out with 4 nM DnaE and 16 nM PykA monomers (4 nM PykA tetramers) or 16 nM CAT, as described in the Section 2.5. Lanes 1 and 2 show the 15 mer and 60 mer oligonucleotide markers, respectively. (**D**) Primer extension assays showing the biochemical effects of PykA and PEPut on the DnaE polymerase activity. Time-course reactions (30, 60, 90, and 120 s, represented schematically from left to right by the rectangular triangles) were carried out with 4 nM DnaE and 16 nM PykA monomers (4 nM PykA tetramer) or 16 nM PEPut, as described in the Section 2.5. Lane 1 shows the position of the 15 mer oligonucleotide marker while the position of the 60 mer oligonucleotide is indicated by the side of the gel. (**E**) Activity assays of the purified full-length PykA and CAT. The assays were carried out as described in Section 2.1.4, PykA activity assay. All assays were carried out in triplicates and the mean reaction rates were plotted against PEP concentration with the error bars showing the standard error.

PykA stimulates the polymerase activity of DnaE in primer extension in vitro assays, implying that a physical interaction between the two proteins mediates this functional effect [5]. In an effort to first identify whether an individual domain, either CAT or PEPut, of PykA mediates this interaction or whether a full-length PykA protein is essential for this interaction, we overproduced and purified the CAT and PEPut domains separately (Figure 2A). We then carried out primer extension in vitro assays using a primed probe constructed from synthetic oligonucleotides to establish whether any one of the individual domains of PykA are capable of exerting a functional effect on the DnaE polymerase activity (Figure 2B). Data confirmed the stimulation of DnaE polymerase activity by PykA, and showed that CAT significantly inhibits this activity whereas PEPut alone does not appear to affect it (Figure 2C,D). This result suggests that CAT mediates the physical and functional interaction with DnaE and that PEPut modulates allosterically the effect of CAT on DnaE.

It was previously shown that PEPut has no metabolic activity per se but modulates the catalytic activity of PykA [5,16]. To confirm this, we compared the PykA and CAT catalytic activities using a standard biochemical assay (see Material and Methods for details). The results showed that CAT alone, when not linked to PEPut, is significantly more active than PykA (Figure 2E), confirming the negative allosteric control of PEPut on PykA catalytic activity.

### 3.2. The CAT Domain Mediates the Physical Interaction with DnaE in a Complex with a Primed Template and PEPut Reduces the Strength of This Interaction

**Figure 3:**
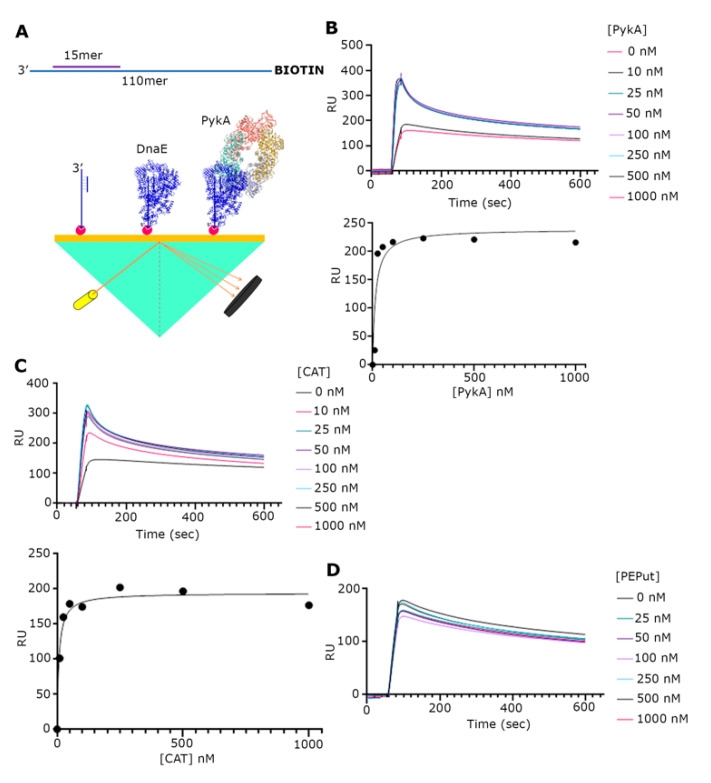
SPR analysis of the PykA-DnaE interaction. (**A**) Schematic diagrams explaining the primed DNA probe that was immobilized on the chip surface and the general SPR set up. (**B**) SPR sensograms with DnaE (35 nM) and increasing concentrations of full-length PykA, as indicated. Underneath the sensograms, the graph shows the fitting of the data to a one site-specific binding curve, using GraphPrism 9. (**C**) SPR sensograms with DnaE (35 nM) and increasing concentrations of CAT, as indicated. Underneath the sensograms, the graph shows the fitting of the data to a one site-specific binding curve, using GraphPrism 9. (**D**) SPR sensograms with DnaE (35 nM) and increasing concentrations of PEPut, as indicated, showing no interaction between DnaE and PEPut.

Although a functional interaction between the PykA and DnaE proteins has been demonstrated, there is still no direct evidence of a physical interaction between the two proteins. To address this issue, we used SPR (Figure 3A). Our initial investigations with just purified DnaE and PykA proteins by immobilizing DnaE on the surface and then adding PykA did not reveal an interaction despite significant efforts (Supplementary Figure S2). One important component that was missing from our set up was DNA; hence, we constructed a primed probe composed of a 30 mer annealed to a 110 mer synthetic oligonucleotide. Next, the probe biotinylated at the 5′-end of the long oligonucleotide was immobilized on the streptavidin-coated surface of a chip (Figure 3A). The initial addition of DnaE at increasing concentrations (0, 10, 35, 87.5, 175, 350, and 700 nM) produced characteristic binding curves indicating a strong binding of DnaE to the probe (Supplementary Figure S3A). The maximum RU values against the DnaE concentration were fitted to a non-linear regression one site-specific binding curve using GraphPad Prism 9, revealed that DnaE binds to the probe with a *K*_d_ = 146.5 nM (R2 = 0.9587) (Supplementary Figure S3B). The subsequent experiments adding DnaE (35 nM) with increasing concentrations of PykA (0, 10, 25, 50, 100, 250, 500, and 1000 nM) produced characteristic binding curves, indicating an interaction between DnaE and PykA (Figure 3B).The maximum RU values of each curve against the (PykA) fitted to a non-linear regression as above, which revealed that the DnaE–PykA interaction has a *K*_d_ = 14.57 nM (R2 = 0.8515), indicating a strong and stable interaction. Similar SPR experiments with the CAT and PEPut domains revealed a strong interaction with CAT (*K*_d_ = 7.33 nM, R^2^ = 0.9672) (Figure 3C), but no interaction with PEPut (Figure 3D). Control SPR experiments confirmed that PykA, CAT, and PEPut do not interact with the DNA probe (Supplementary Figure S4). Thus, we conclude from these data that the physical interaction of PykA with DnaE is mediated by CAT and that, because of our inability to detect an interaction between the two proteins in the absence of DNA, this interaction is DNA-dependent. The two-fold higher affinity of CAT (*K*_d_ = 7.33 nM) over PykA (*K*_d_ = 14.57 nM) for the DnaE-DNA complex further highlights the importance of PEPut in allosterically regulating PykA replication functions.

### 3.3. The TSH Motif of PEPut Impacts the Catalytic Activity of PykA but Not Its Effect on DnaE Activity

**Figure 4:**
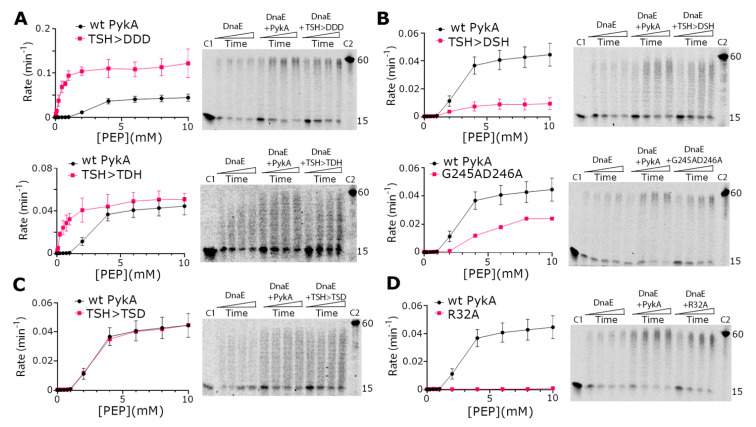
Biochemical characterization of PykA mutant proteins. PykA activity assays were carried out as described in ‘Materials and Methods’ and different mutations were found to increase (panel **A**), decrease (panel **B**), have no effect (panel **C**), or abolish (panel **D**) the PykA activity. All PykA activity assays were performed in triplicates and the mean rates were plotted against PEP concentration using GraphPad Prism 9, with the error bars showing the standard error. All PykA mutants were tested for the ability to stimulate the primer extension activity of DnaE and were all found able to stimulate DnaE, just like the wild-type PykA did. Primer extension assays were carried out as described in Section 2.5 with 4 nM DnaE and 16 nM PykA monomers (4 nM tetramers) in time courses 30, 60, 90, and 120 s, which are represented schematically from left to right by the rectangular triangles. C1 and C2 represent the annealed 15 mer primer and the maximum length 60 mer product of the primer extension reaction, respectively.

To acquired insights concerning the involvement of PEPut in PykA replication and metabolic functions, PykA proteins mutated in the TSH motif of PEPut were purified. This mutagenesis study was motivated by previous data showing that TSH mutations have different and contrasted effects on replication initiation and PykA catalytic activities [5], and that this motif is phosphorylated in other metabolic enzymes to ensure catalytic and regulatory functions (see above). In the purified mutant proteins, the three residues of the TSH motif were replaced individually or collectively by a D for mimicking phosphorylation. We also purified two proteins that were mutated in the catalytic site of PykA as controls. These mutations (R32A and G245AD246A) stimulate replication elongation in vivo and strongly reduce PykA activity [5]. The results showed that all mutant proteins stimulated the DnaE polymerase activity just like the wild-type PykA protein, suggesting that none of the tested mutations disrupt the PykA-DnaE interaction, its effect on DnaE activity, and its modulation by PEPut (Figure 4). In contrast, the catalytic activity of PykA is differently affected by the mutations. While the PEPut TSH > DDD and TSH > TDH mutations increase PykA activities, the TSH > DSH and TSH > TSD mutations decrease it or have no effect, respectively. Mutations in the catalytic site either reduced PykA activity (G245AD246A) or resulted in a catalytically dead protein (R32A). Hence, PykA catalytic activity is strongly modulated by PEPut, with T and S phosphorylation having opposite effects (inhibition and stimulation, respectively). Moreover, while the CAT and PykATSH > DDD proteins exhibit similar, very strong, catalytic activities, they oppositely impact DnaE activity (inhibition and stimulation, respectively) (compare the top panels of Figure 4A with Figure 2C,E). This shows that, whereas the TSH > DDD mutation mimics PEPut deletion in the metabolic assay, it does not do so in the replication assay. This suggests that the TSH > DDD mutation abrogates the metabolic but not the replication function of PEPut. Overall, these results highlight the complex and somewhat distinct functions played by the PEPut domain in modulating the effect of PykA on DnaE activity and PykA catalytic activity.

### 3.4. PEPut and Cat Mutations Impact Origin and Replisome Localization

**Figure 5:**
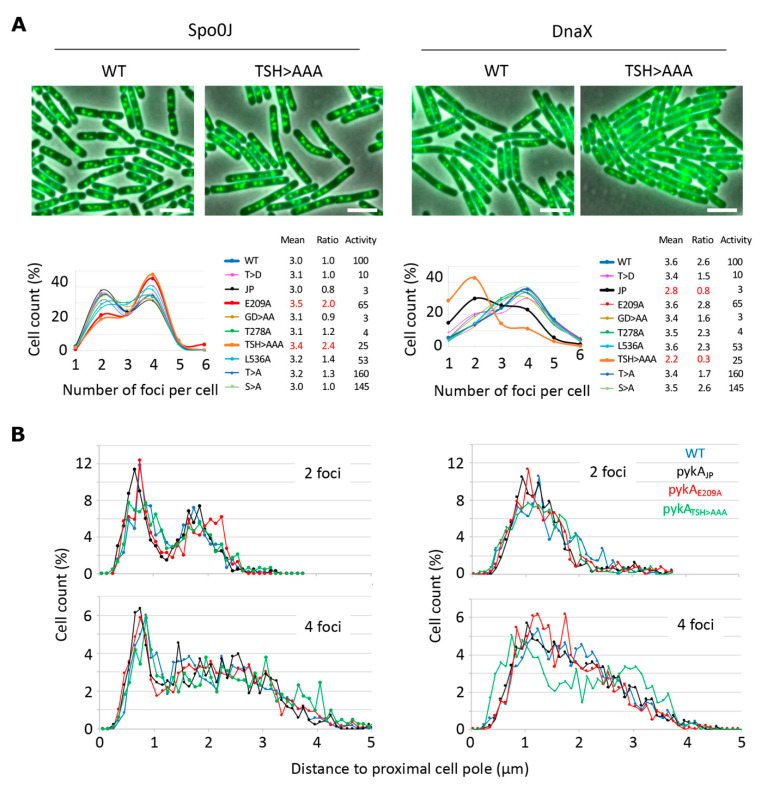
Pattern of Spo0J-GFP and DnaX-GFP foci in wild-type cells and *pykA* mutants. Cells encoding the GFP fusion proteins were grown in MC and foci distribution was analyzed by microscopy at OD_650 nm_ = 0.15 in populations of about 2500 bacteria. (**A**) Proportion of cells with 1 to 6 foci. Top row: representative overlays of the phase contrast and GFP images of wild-type (WT) and *pykA_TSH>AAA_* (TSH > AAA) cells. Scale bar: 4 µm. Bottom row: Distribution of cells with n Spo0J-GFP or DnaX-GFP foci. Mean: Mean number of foci per cell. Ratio: Number of cells with 4 over 2 foci. Activity: Pyruvate kinase activity (%) [5]. Mutant proteins are described in the Supplementary Table S1. (**B**) Distance pole-foci along the long cell axis (using as reference the pole closest to the first foci). Note that the mean cell size of pykA_E209A_, pykA_T537A_, pykA_T537D_, and pykA_TSH>AAA_ cells varies by <5% compared to the wild-type strain (4.1 µm; 400 cells analyzed by strain; Spo0J context).

The finding that PykA physically interacts with DnaE suggests that this metabolic enzyme can be recruited to the orisome and replisome to influence replication initiation, and elongation. Unfortunately, and despite significant efforts, we were unable to clearly localize PykA-fluorescent protein fusions by epi-fluorescence microscopy [5]. In an attempt to find indirect evidence of PykA recruitment at orisomes and replisomes, we analyzed *oriCs* and replication fork distribution in live wild-type and *pykA* cells. The visualization of *oriCs* and replication forks was carried out in cells encoding the Spo0J-GFP or DnaX-GFP protein fusions, respectively [32], and the foci distribution was analyzed in wild-type and representative CAT and PEPut mutants. Cells were grown in the MC gluconeogenic medium (malate + casein pancreatic hydrolysate), which was previously extensively used to analyze PykA metabolic and replication functions [5]. Most of the mutants (14/18) exhibited an average number of foci per cell and a ratio of cells with four over two foci similar to the wild-type strain (Figure 5A). However, these parameters significantly increased for *oriCs* foci (Spo0J-GFP context) in the Cat (PykA_E209A_) and PEPut (PykA_TSH>AAA_) mutants and notably decreased for replication forks foci (DnaX-GFP context) in the Cat (PykA_JP_) and PEPut (PykA_TSH>AAA_) mutants. Moreover, changes in foci positioning along the long cell axis were found in one mutant (in *pykA_TSH>AAA_* cells with four DnaX-GFP foci, Figure 5B).

Theoretically, changes in foci patterns may be caused by changes in cell division. In *B. subtilis*, this process depends on pyruvate and the UDP-glucose concentration [33,34]. Given that PykA produces pyruvate from PEP in the last reaction of glycolysis and that UDP-glucose is a derivative of the first metabolite of glycolysis (glucose-6P), one can hypothesize that PykA mutations change foci patterns by altering pyruvate and/or UDP-glucose concentration and hence, cell division. However, the metabolome analysis argues against this hypothesis [5] as well as the lack of covariation between PykA activity and foci patterns (Figure 5A, inserted table). Strikingly, we found wild-type numbers of foci per cell and wild-type ratios of cells with four over two foci in strains encoding a PykA activity ranging from 3 to 165% of the parental strain, while these parameters were altered in mutants with a PykA activity of 3, 25, and 65%. Moreover, no significant cell size changes (<5%) were found in representative *pykA* mutants compared to the wild-type strain (Figure 5). Collectively, our results suggest that CAT and PEPut are important for the spatial localization of origins and replisomes, independent of their function in PykA catalytic activity. We suggest that these results provide indirect evidence for PykA recruitment at orisomes and replisomes.

## 4. Discussion

Despite our exquisite knowledge of the molecular mechanisms that underpin replication and cellular metabolism, our understanding on how these two of the most fundamental functions of life regulate each other’s activities is limited. Recently, PykA has emerged as a new family of cross-species replication control regulators that drives the metabolic control of replication through a mechanism involving regulatory determinants of PykA catalytic activity, namely, CAT-substrate interactions, CAT-PEPut interaction, and PEPut TSH phosphorylation [5]. The CAT domain of PykA was shown to stimulate the replication fork speed whereas the PEPut domain of PykA was shown to act as a nutrient-dependent inhibitor of replication initiation [5]. Purified PykA forms an active, stable tetramer that stimulates the polymerase activity of DnaE, suggesting a functional interaction, but no direct protein–protein interaction between DnaE and PykA has been demonstrated to date [5]. Here, we show that PykA physically interacts with DnaE via a CAT–DnaE interaction when DnaE is bound to a primed DNA template and that PEPut modulates this interaction, the effect of PykA on DnaE activity, and the PykA catalytic activity.

The SPR data presented in this paper uncover a physical protein–protein interaction between DnaE and PykA. This interaction occurs when the polymerase is bound to a primed template and is mediated by the CAT domain of PykA. In contrast, the purified PEPut domain has no effect on DnaE activity and does not show any signs of a physical interaction with the DnaE. Interestingly, PykA and CAT have distinct effects on DnaE: while PykA stimulates DnaE activity CAT inhibits it, and the interaction of the DnaE-DNA complex with PykA is weaker than with CAT (*K*_d_ = 14.57 versus 7.33 nM, respectively). As the two proteins differ by the presence/absence of PEPut, and as PEPut interacts with CAT through hydrogen bonding [15,29], the distinct effects of PykA and CAT on DnaE may result from some allosteric changes induced by PEPut. If so, this may assign a regulatory function of PEPut on the replication moonlighting activity of PykA. Unfortunately, we were unable to identify the residues that were important for this regulatory function, as the replication phenotypes of purified PykA mutants (two were mutated in CAT and four in the TSH motif of PEPut) were indistinguishable from that of the native protein. This is in contrast to the in vivo data, which showed that the CAT mutations used here stimulate the rate of replication forks while those in the TSH motif abrogate or leave intact the PEPut-driven inhibitory activity of initiation [5]. Clearly, the simplified assay used here does not recapitulate the in vivo situation and needs to be significantly improved to approach the complexity of live cells.

A parallel study was carried out to further characterize the factors regulating the PykA catalytic activity. On its own, the CAT domain exhibits a significantly increased activity compared to the full-length PykA protein. As most of the bacterial PykA proteins lack PEPut [15], and as the related PEPut-containing the *Geobacillus stearothermophilus* protein forms an active tetramer with and without its PEPut domain [29], our results suggest that the difference in CAT and PykA activities results from a PEPut-mediated allosteric regulation of PykA activity, as proposed previously [5,15]. This notion is supported by our data, showing that the PykA catalytic activity is either increased (TSH > TDH and TSH > DDD), impaired (TSH > DSH), or left intact (TSH > TSD) in the mutants of the TSH motif of PEPut. Although these results are in good agreement with our previous study [5], some apparent contradictions were found: the TSH > TDH and TSH > DDD mutants were found to be highly active here but they were poorly active in the previous study. We envision that these differences result from differences in the experimental set up (standard assay versus crude extract assay), indicating that the standard assay needs to be complemented with additional factors to fully recapitulate the complexity of the allosteric regulation of PykA activity by PEPut.

The detection of a strong interaction between PykA and DnaE when bound to a primed DNA template is an important discovery toward the molecular understanding of the metabolic control of replication. Indeed, this result indicates that PykA is recruited by DnaE at sites of DNA synthesis and that the thousands of cytosolic PykA molecules cannot trap the tens of free (not DNA-bound) DnaE proteins far away from replication sites. The PykA recruitment at sites of DNA synthesis is likely highly dynamic, as DnaE is frequently recruited to and released from replication machineries to extend RNA primers during replication initiation and lagging strand synthesis [30,31,35,36,37]. This implies that the PykA-DnaE interactions are incessantly dissociated and reconstituted, allowing new PykA molecules to be recruited at replication machineries to ensure a highly dynamic modulation of the replication rate with metabolism. Obviously, this hypothesis needs to be substantiated by additional data, including direct evidence of PykA recruitment at origins and replication forks in live cells. Interestingly, the microscopy data presented here provide indirect support for this recruitment, showing that the CAT and PEPut domains are important for a proper spatial localization of origins and replication forks, independent of their functions in PykA catalytic activity.

Our inability to detect an interaction between the PEPut domain and DnaE raises the question of how PEPut modulates replication initiation. Several hypotheses can be put forward to address this issue. First, it can be considered that our assay, which monitors DNA synthesis and not DNA initiation, does not contain structures such as supercoiled DNA and melted double-strand DNA needed for PykA recruitment via a DnaE-PEPut interaction. Alternatively, it may be that the PEPut domain interacts with some other components of the *B. subtilis* orisome (e.g., DnaA, DnaC, etc.…) via an as yet unidentified physical interaction. It may also be that only the CAT domain physically interacts with DnaE (and perhaps other replication proteins) with the PEPut domain controlling the replication moonlighting functions of CAT on initiation or elongation. This control could depend on the growth conditions. As previously shown, suggesting that the PEPut initiation function is turned on in neoglucogenic media and off in glycolytic media [5].

A long history of investigations suggest that the metabolism-replication links described here and in our previous work [5] are more the tip of an iceberg than the exception. In *B. subtilis*, comprehensive studies uncovered a toolbox of intermingled metabolism-replication links that temporalizes replication in the cell cycle in a nutritional-dependent manner. This toolbox comprises on one side, reactions ensuring the 3C part of glycolysis (including PykA) and the downstream pyruvate metabolism, and, on the other side, the replication enzymes DnaC, DnaG, and DnaE [3,4,38]. Similar results were found in *E. coli* [39,40,41] and possibly *Caulobacter crescentus* [42]. In *E. coli*, evidence for the modulation of initiation by metabolites (acetyl-CoA and cAMP) was also provided [43,44]. In eukaryotes, the timing of the origin firing depends on an increase in acetyl-CoA, which promotes histone acetylation [45]. This increase is geared by a metabolic cycle in yeast and by nuclear forms of the ATP-citrate lyase and PDH complexes in mammalian cells [46,47,48]. Moreover, the glyceraldehyde-3-phosphate dehydrogenase (GAPDH) and the lactate dehydrogenase (LDH) enter the nucleus to induce histone H2B production and promote S-phase progression [49,50]. Another metabolic enzyme, the phosphoglycerate kinase PGK, interacts with the protein kinase CDC7 in the nucleus to stimulate replication initiation by enhancing the CDC7-mediated activation of the replicative MCM helicase [51]. Additionally, the impaired expression of genes of the central carbon metabolism delays the entry of human fibroblasts into the S phase or decreases the number of cells in this phase [52,53,54]. Finally, PGK, GAPDH, and LDH modulate the activity of the three eukaryotic replicative polymerases (Polα, Polε, and Polδ) in vitro [55,56,57]. Collectively, these results suggest that DNA replication is under a metabolic control geared by determinants of the central carbon metabolism throughout the evolution tree. Since Schaechter’s seminal studies in *Salmonella typhimurium* in 1958, it is now well-established that this control compartmentalizes DNA synthesis in the cell cycle in a wide range of nutritional conditions in bacteria, while it directs the entry and progression of the S phase in the reduction phase of an oscillating redox metabolic cycle in eukaryote cells [58,59,60,61]. By extrapolating knowledge on PykA moonlighting activities in DNA replication, we proposed that the metabolic control of replication from bacteria to eukaryote is orchestrated by a mechanism in which metabolites and proteins of central carbon metabolism that signal and sense nutritional stimuli for regulating cellular metabolism, ensure moonlighting activities to convey this metabolic information to the replication machinery for coordinating replication to metabolism. As the metabolic control of replication plays an important role in the overall replication rate [5], and as failures in this control cause chromosomal lesions (double-strand DNA breaks and nucleotide misincorporation) increasing the risk of genetic diseases, such as cancer [6,7,8,9], we propose that metabolic changes underpinning the Warburg effect and disrupting the metabolic control of replication may form an additional, intrinsic root cause of genetic instability and cancer initiation.

## 5. Conclusions

Our report provides novel molecular insights into the metabolic control of DNA replication in *B. subtilis*. It suggests that (i) this control depends on a direct physical interaction between the CAT domain of PykA and the DNA polymerase DnaE, which is essential for replication initiation and lagging strand synthesis; (ii) this interaction occurs at primed sites, allowing PykA recruitment within the orisome and replisome; and (iii) the PEPut domain of PykA regulates, at least in part, the moonlighting replication functions of PykA and is also a key regulator of PykA catalytic activity, thereby connecting replication to metabolism.

## Figures and Tables

**Table 1:** A list of the oligonucleotides used to create the PykA mutations and the CAT construct. The mutated sequences are shown in bold small caps.

PykA R32A Fwd	GAACGTGGCT**gcA**TTAAACTTTTC
PykA R32A Rev	ATTCCTGACTCCATTAATTTC
PykA G245A D246A Fwd	GGTTGCACGC**gcagca**TTAGGTGTGG
PykA G245A D246A Rev	ATTAAGCCGTCAGACACTTC
PykA TSH T537D Fwd	AGGCGGTTTG**gat**AGCCATGCTG
PykA TSH T537D Rev	TCTTCTGTAATAAGAGCAGAC
PykA TSH S538D Fwd	CGGTTTGACT**gat**CATGCTGCGG
PykA TSH S538D Rev	CCTTCTTCTGTAATAAGAGC
PykA TSH H539D Fwd	TTTGACTAGC**gat**GCTGCGGTAG
PykA TSH H539D Rev	CCGCCTTCTTCTGTAATAAG
FWD PykA_TSH DDD	CGGTTTGGAT**gatg**ATGCTGCGGTAG
REV PykA_TSH DDD	CCTTCTTCTGTAATAAGAGC
PykA CAT fwd	TAATAACATTGGAAGTGGATAAC
PykA CAT rev	GCCGACAGTATGAACCTTC

## Data Availability

All data are contained within the article.

## Supplementary Materials

The following supporting information can be downloaded at: https://www.mdpi.com/article/10.3390/life13040965/s1, Figure S1: The plasmid map of the p2CT-*pykA* expression vector. Figure S2: SPR data showing no interaction between DnaE (immobilized on the chip surface) and PykA. Figure S3: SPR sensograms of increasing concentrations of DnaE; Figure S4: Control SPR experiments with increasing concentrations of PykA, CAT and PEPut; Table S1: Strains.
