# Supplementary material for: The Replicative DnaE Polymerase of Bacillus subtilis Recruits the Glycolytic Pyruvate Kinase (PykA) When Bound to Primed DNA Templates
Source: Life (Basel). 2023 Apr 7;13(4):965. doi: 10.3390/life13040965 (PMC10143966; doi:10.3390/life13040965)
Supplement: Supplementary file 1 [file life-13-00965-s001.zip › life-2283704-supplementary.pdf]

Supplementary Information

The Replicative DnaE Polymerase of *Bacillus subtilis* Recruits the Glycolytic Pyruvate Kinase (PykA) When Bound to Primed DNA Templates

Holland, Alexandria [1], Pitoulias, Matthaios [1], Soultanas, Panos [1,*] and Janniere, Laurent [2,*]

1 Biodiscovery Institute, School of Chemistry, University of Nottingham, Nottingham NG7 2RD, UK
2 Génomique Métabolique, Genoscope, Institut François Jacob, CEA, CNRS,

Université Evry, Université Paris-Saclay, 91057 Evry, CEDEX, France

* Correspondence: panos.soultanas@nottingham.ac.uk (P.S.); laurent.janniere@univ-evry.fr (L.J.)

Supplementary Tables

Table S1 (strains)

Types of strains	Genotype	Main phenotypes	Name	Context	Source	Obtained by transformation with:[1]
Parental strains	*trpC2*	wild-type, cured of prophages PBSX, SPβ and SKIN	TF8A	168	[3]	
	dnaX-gfpmut2-spc	DnaX-GFP, SpcR	BTS8	PY79	Lyle A Simmons	
	spo0J-gfpmut2-spc	Spo0J-GFP, SpcR	LAS26	PY79	Lyle A Simmons	
	pykA$_{E209A}$*-tet*	Mutation E209A in the Cat domain, TetR	DGRM1046	TF8A	[5]	
	pykA$_{GD245\text{-}246AA}$*-tet*	GD245-246AA mutation in the Cat domain of PykA, TetR	DGRM1097	TF8A	[5]	
	pykA$_{T278A}$*-tet*	T278A mutation in the Cat domain of PykA, TetR	DGRM1047	TF8A	[5]	
	pykAJP-prm	Deletion of 27 amino acids (208-234) in the Cat domain of PykA, PhlR	DGRM24-Phl	TF8A	[3]	
	pykA$_{L>A}$*-tet*	Mutation L536A in the PEPut domain, TetR	DGRM1048	TF8A	[5]	
	pykA$_{T>A}$*-tet*	Mutation T537A in the PEPut domain, TetR	DGRM299	TF8A	[5]	
	pykA$_{S>A}$*-tet*	Mutation S538A in the PEPut domain, TetR	DGRM303	TF8A	[5]	
	pykA$_{TSH>AAA}$*-tet*	Mutation TSH537-539AAA in the PEPut domain, TetR	DGRM302	TF8A	[5]	
	pykA$_{T>D}$*-tet*	Mutation T537D in the PEPut domain, TetR	DGRM1018	TF8A	[5]	
Spo0J-GFP and DnaX-GFP strains	*dnaX-gfpmut2-spc*	DnaX-GFP, SpcR	DGRM1100	TF8A	This work	BTS8 → TF8A (Sp)
	spo0J-gfpmut2-spc	Spo0J-GFP, SpcR	DGRM1101	TF8A	This work	LAS26 → TF8A (Sp)
	pykA$_{T>D}$*-tet dnaX-gfpmut2-spc*	T537D mutation in the PEPut domain of PykA, DnaX-GFP, TetR, SpcR	DGRM1126	TF8A	This work	DGRM1100 → DGRM1018 (Sp)
	pykA$_{T>D}$*-tet spo0J-gfpmut2-spc*	T537D mutation in the PEPut domain of PykA, Spo0J-GFP, TetR, SpcR	DGRM1127	TF8A	This work	DGRM1101 → DGRM1018 (Sp)
	pykA$_{E209A}$*-tet dnaX-gfpmut2-spc*	E209A mutation in the Cat domain of PykA, DnaX-GFP, TetR, SpecR	DGRM1150	TF8A	This work	DGRM1100 → DGRM1046 (Sp)
	pykA$_{E209A}$*-tet spo0J-gfpmut2-spc*	E209A mutation in the Cat domain of PykA, Spo0J-GFP, TetR, SpecR	DGRM1151	TF8A	This work	DGRM1101 → DGRM1046 (Sp)
	pykA$_{GD245/6AA}$*-tet dnaX-gfpmut2-spc*	GD245/6AA mutation in the Cat domain of PykA, DnaX-GFP, TetR, SpecR	DGRM1152	TF8A	This work	DGRM1100 → DGRM1097 (Sp)
	pykA$_{GD245/6AA}$*-tet spo0J-gfpmut2-spc*	GD245/6AA mutation in the Cat domain of PykA, Spo0J-GFP, TetR, SpecR	DGRM1153	TF8A	This work	DGRM1101 → DGRM1097 (Sp)
	pykA$_{T278A}$*-tet dnaX-gfpmut2-spc*	T278A mutation in the Cat domain of PykA, DnaX-GFP, TetR, SpecR	DGRM1154	TF8A	This work	DGRM1100 → DGRM1047 (Sp)
	pykA$_{T278A}$*-tet spo0J-gfpmut2-spc*	T278A mutation in the Cat domain of PykA, Spo0J-GFP, TetR, SpecR	DGRM1155	TF8A	This work	DGRM1101 → DGRM1047 (Sp)
	pykAJP-prm dnaX-gfpmut2-spc	Deletion of 27 amino acids (208-234) in the Cat domain of PykA, DnaX-GFP, PhlR, SpecR	DGRM1156	TF8A	This work	DGRM1100 → DGRM24-Phl (Sp)
	pykAJP-prm spo0J-gfpmut2-spc	Deletion of 27 amino acids (208-234) in the Cat domain of PykA, Spo0J-GFP, PhlR, SpecR	DGRM1157	TF8A	This work	DGRM1101 → DGRM24-Phl (Sp)
	pykA$_{L536A}$*-tet dnaX-gfpmut2-spc*	L536A mutation in the PEPut domain of PykA, DnaX-GFP, TetR, SpecR	DGRM1158	TF8A	This work	DGRM1100 → DGRM1048 (Sp)
	pykA$_{L536A}$*-tet spo0J-gfpmut2-spc*	L536A mutation in the PEPut domain of PykA, Spo0J-GFP, TetR, SpecR	DGRM1159	TF8A	This work	DGRM1101 → DGRM1048 (Sp)
	pykA$_{TSH>AAA}$*-tet dnaX-gfpmut2-spc*	TSH537-539AAA mutation in the PEPut domain of PykA, DnaX-GFP, TetR, SpecR	DGRM1160	TF8A	This work	DGRM1100 → DGRM302 (Sp)
	pykA$_{TSH>AAA}$*-tet spo0J-gfpmut2-spc*	TSH537-539AAA mutation in the PEPut domain of PykA, Spo0J-GFP, TetR, SpecR	DGRM1161	TF8A	This work	DGRM1101 → DGRM302 (Sp)
	pykA$_{T>A}$*-tet dnaX-gfpmut2-spc*	T537A mutation in the PEPut domain of PykA, DnaX-GFP, TetR, SpecR	DGRM1162	TF8A	This work	DGRM1100 → DGRM299 (Sp)
	pykA$_{T>A}$*-tet spo0J-gfpmut2-spc*	T537A mutation in the PEPut domain of PykA, Spo0J-GFP, TetR, SpecR	DGRM1163	TF8A	This work	DGRM1101 → DGRM299 (Sp)
	pykA$_{S>A}$*-tet dnaX-gfpmut2-spc*	S538A mutation in the PEPut domain of PykA, DnaX-GFP, TetR, SpecR	DGRM1164	TF8A	This work	DGRM1100 → DGRM303 (Sp)
	pykA$_{S>A}$*-tet spo0J-gfpmut2-spc*	S538A mutation in the PEPut domain of PykA, Spo0J-GFP, TetR, SpecR	DGRM1165	TF8A	This work	DGRM1101 → DGRM303 (Sp)

1: X → Y indicates that strain Y was transformed with DNA from source X using the selection indicated in brakets.

Supplementary Figures

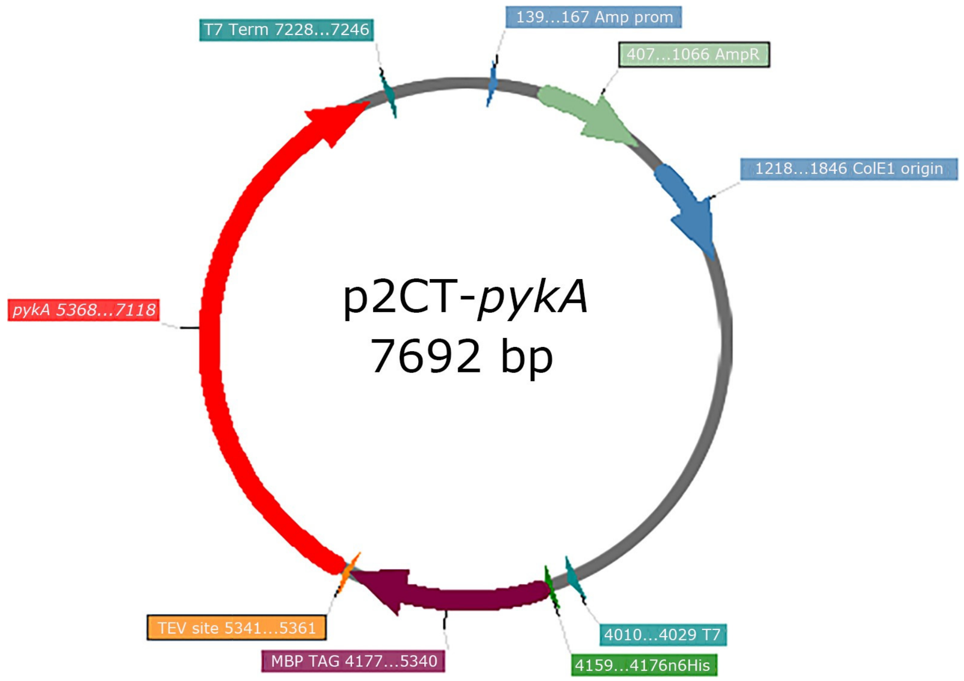

Supplementary Figure S1

The plasmid map of the p2CT-*pykA* expression vector. The p2CT backbone is commercially available via Addgene (https://www.addgene.org/83487/). This vector produces an N-terminally His-tagged/MBP PykA protein with the His-MBP tag removable by proteolysis using the TEV protease. The p2CT-PEPut production vector is identical to the p2CT-*pykA* with the PEPut fragment cloned instead of the *dnaE* gene at exactly the same position.

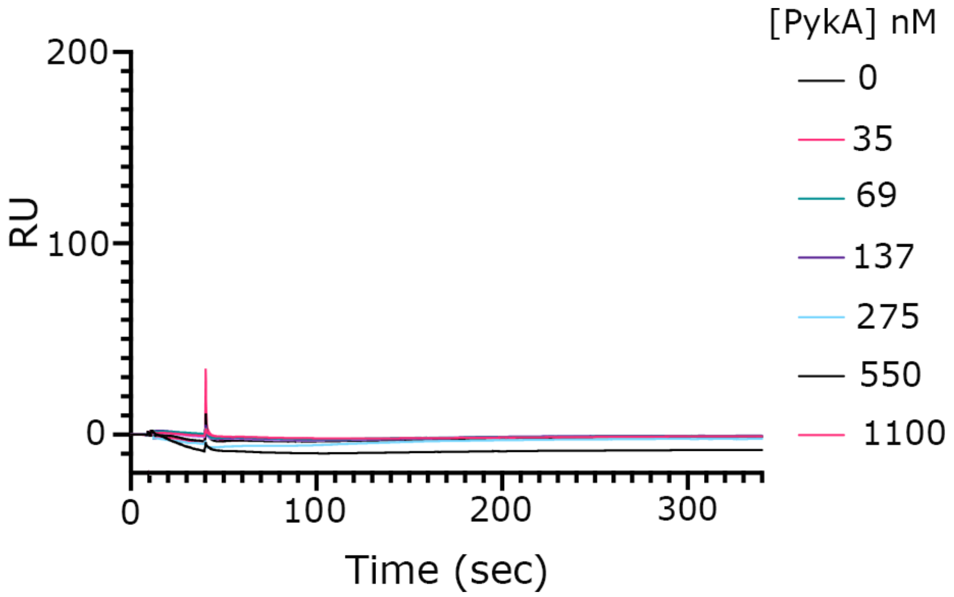

Supplementary Figure S2

SPR data showing no interaction between DnaE (immobilized on the chip surface) and PykA. The Biacore instrument was running with a buffer (20 mM sodium acetate pH 5.0, 0.005% (w/v) Tween 20), which was filtered through a 0.22 µm filter and degassed before the sensor chip C1 was docked into the instrument. Its surface was washed with three injections of 10 µL 0.1 M glycine-NaOH pH 12.0 containing 0.3% (w/v) Triton X-100 at 10 µL/min. Then, a prime run was performed using the running buffer to remove the Triton X-100 from the tubing and the sensor chip. The carboxyl groups on the sensor chip were activated with a mixture of 0.2 M EDC (1-Ethyl-3-[3-dimethylaminopropyl]carboiimide Hydrochloride) and 0.05 M NHS (*N*-hydroxysuccinimide) to give reactive succinimide esters. A total of 70 µL of the mixture was loaded onto two channels of the chip (one for the control without the ligand and the other for the immobilized ligand) at 10 µL for 7 min. To bind the DnaE protein to the reactive esters, 20 mM sodium acetate pH 5.0, 0.005% (w/v) Tween 20 was passed through the channels. Then, successive injections of 10 µL of 1 nM DnaE were performed at 10 µL/min for 1 min in order to reach approximately 200 RU on one of the channels, while the second channel was injected with the same buffer that contained no DnaE protein as a control. Finally, the channels were injected and equilibrated with 40 mM Tris HCl pH 8.0, 150 mM NaCl, 2 mM $MgCl_2$, and 0.005% (w/v) Tween 20 prior to the loading of the analyte (PykA).

PykA (10 µL) was injected at increasing concentrations at 2 µL/min for approximately 5 min. The data were exported to GraphPad 9.0 and plotted as RU versus (PykA) after subtracting the control data from the experimental data. There was no detectable interaction between DnaE and PykA and all the sensograms were flat lines superposed on top of each other on top of the X-axis.

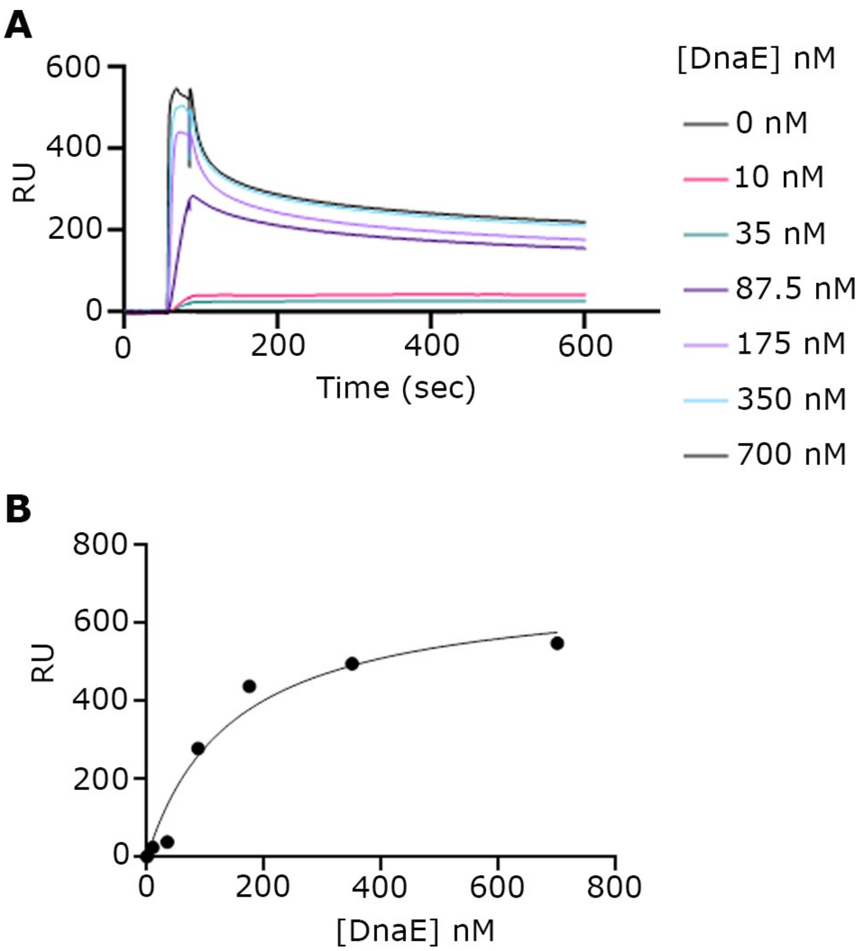

Supplementary Figure S3

A. SPR sensograms of increasing concentrations of DnaE, as indicated. Clear concentration-dependent binding of DnaE to the DNA probe immobilized onto the surface of the tip is evident.

B. The SPR data were fitted to a one site-specific binding curve using GraphPad Prism 9, revealing that DnaE binds strongly to the DNA probe with a K_d=146.5 nM (R^2=0.9587).

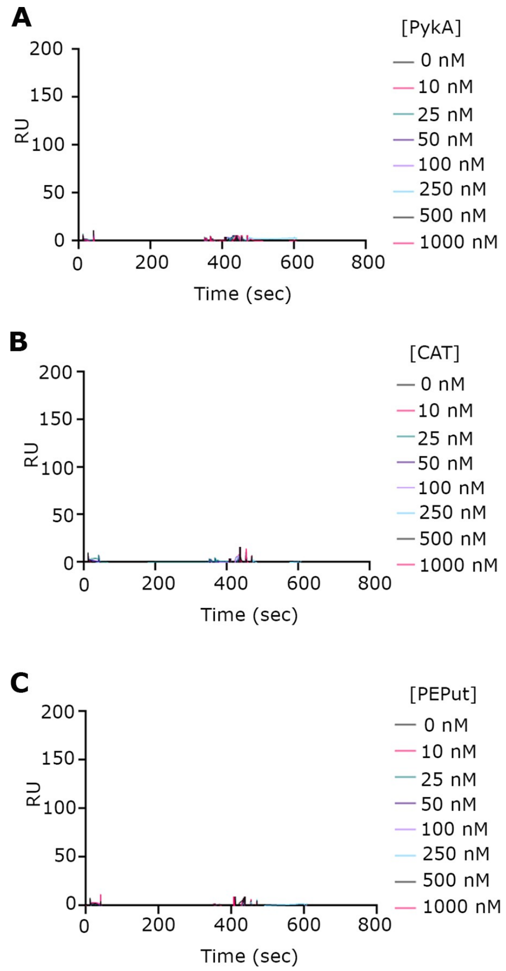

Supplementary Figure S4

Control SPR experiments with increasing concentrations of PykA (**A**), CAT (**B**), and PEPut (**C**), as indicated, showing that these proteins do not bind to the DNA probe that is immobilized on the surface of the SPR chip. The sensograms reveal no binding as they are all flat and superposed on the X-axes.
